# A Novel Three-Choice Touchscreen Task to Examine Spatial Attention and Orienting Responses in Rodents

**DOI:** 10.1523/ENEURO.0032-20.2021

**Published:** 2021-07-09

**Authors:** Faraj L. Haddad, Maryam Ghahremani, Cleusa De Oliveira, Ella E. Doornaert, Kevin D. Johnston, Stefan Everling, Susanne Schmid

**Affiliations:** 1 Graduate Program in Neuroscience, The University of Western Ontario, London, Ontario, N6A3K7, Canada; 2 Anatomy and Cell Biology, Schulich School of Medicine and Dentistry, The University of Western Ontario, London, Ontario, N6A3K7, Canada; 3 Department of Physiology and Pharmacology, The University of Western Ontario, London, Ontario, N6A3K7, Canada; 4 Robarts Research Institute, The University of Western Ontario, London, Ontario, N6A3K7, Canada; 5 Department of Psychology, The University of Western Ontario, London, Ontario, N6A3K7, Canada

**Keywords:** orienting behavior, rat, superior colliculus, touch-screen

## Abstract

Mammalian orienting behavior consists of coordinated movements of the eyes, head, pinnae, vibrissae, or body to attend to an external stimulus. The present study aimed to develop a novel operant task using a touch-screen system to measure spatial attention. In this task, rats were trained to nose-poke a light stimulus presented in one of three locations. The stimulus was presented more frequently in the center location to develop spatial attention bias toward the center stimulus. Changes in orienting responses were detected by measuring the animals’ response accuracy and latency to stimuli at the lateral locations, following reversible unilateral chemogenetic inactivation of the superior colliculus (SC). Additionally, spontaneous turning and rotation behavior was measured using an open-field test (OFT). Our results show that right SC inactivation significantly increased the whole body turn angle in the OFT, in line with previous literature that indicated an ipsiversive orientating bias and the presence of contralateral neglect following unilateral SC lesions. In the touch screen orienting task, unilateral SC inactivation significantly increased bias toward the ipsilateral side, as measured by response frequency in various experimental conditions, and a very large left-shift of a respective psychometric function. Our results demonstrate that this novel touchscreen task is able to detect changes in spatial attention and orienting responses because of e.g. experimental manipulations or injury with very high sensitivity, while taking advantage of the touch screen technology that allows for high transferability of the task between labs and for open-source data sharing through https://www.mousebytes.ca.

## Significance Statement

Touch-screen rodent testing is a novel translational method of behavioral testing that is more comparable to test batteries used in humans, such as the Cambridge Neuropsychological Test Automated Battery (CANTAB). Its standardized approach in closed boxes allows for better comparability of data between labs and for open-source data sharing at the affiliated platform https://www.mousebytes.ca. The goal of this study was to expand the toolbox for touch-screen boxes to investigate orienting behavior and spatial attention. Unilateral reversible chemogenetic inhibition of the SC revealed an ipsiversive orientating bias and the presence of neglect-like effects for contralateral visual stimuli, demonstrating that this novel task is highly sensitive to detect disruptions of spatial attention associated with psychiatric disorders, brain injury, or experimental manipulations.

## Introduction

Orienting behavior in mammals consists of highly coordinated movements of the eyes, head, pinnae, vibrissae, and body toward salient sensory stimuli. Sensory information relevant to spatial orienting, such as visual stimulus location, is represented topographically in the superior colliculus (SC) across a wide range of vertebrate species (Gaither and Stein, 1979; May, 2006). The SC is a laminar midbrain structure that is critical for the generation of orienting behaviors, serving the goal of aligning the sensory apparatus of an animal with objects of interest in the surrounding environment. Consistent with the evolutionarily highly conserved nature of this structure, SC lesions result in severe orienting impairments in a range of vertebrate species as diverse as tree shrews (Casagrande and Diamond, 1974), cats (Sprague and Meikle, 1965), and nonhuman primates (Schiller et al., 1987). Along the same line of evidence, electrical microstimulation of the SC has revealed a topographic organization of orienting behaviors. In the rhesus macaque, stimulation of rostral regions of the SC evokes small amplitude contraversive saccades (Robinson, 1972), while stimulation of the caudal SC evokes large amplitude contraversive saccades and head movements (Robinson, 1972; Freedman et al., 1996; Corneil et al., 2002). Saccades are rapid eye movements common to primate species that form a considerable part of their orienting behavior by moving their fovea to the visual stimuli of interest. Although rodents do not possess a well-defined fovea to produce the same kind of saccades, electrical stimulation of the SC in rodents has evoked contraversive movements of the eyes and coordinated eye, head, pinnae, vibrissae, and whole-body circling (McHaffie and Stein, 1982; Northmore et al., 1988). Unilateral lesions of the SC in rodents result in two classic changes in orienting behavior: a tendency to circle in the direction of the lesioned SC (ipsilesional circling), and an inability to localize relevant stimuli in the hemifield contralateral to the lesioned SC (contralesional neglect; Sprague and Meikle, 1965; Schneider, 1969; Kirvel et al., 1974; Dean and Redgrave, 1984; Tehovnik, 1989; Felsen and Mainen, 2008; Duan et al., 2015; Kopec et al., 2015). The former deficit has been characterized as a failure of motor implementation of orienting, while the latter has been ascribed to changes in spatial attention, i.e., a failure of visual selection of relevant information akin to visual neglect (Sinnamon and Garcia, 1988; Krauzlis et al., 2013). Contraversive neglect has typically been evaluated by presenting animals with various visual stimuli along the edges of their visual field or in operant testing paradigms that require orienting toward different stimuli to receive a reward (Schneider, 1967, 1975; Sinnamon and Garcia, 1988; Felsen and Mainen, 2008; Stubblefield et al., 2013; Kopec et al., 2015). More recently, touchscreen testing platforms have become available for rodent studies in an attempt to increase translation from rodents to human experimental tasks. Behavioral testing with touch screens is more directly comparable to test batteries used in humans, such as the Cambridge Neuropsychological Test Automated Battery (CANTAB): it uses the same types of stimulus materials (objects and locations on a computer screen), and the same types of responses (responses directly to the stimuli on the screen using a touchscreen apparatus), along with precise control of the timing and identity of visual stimuli (Bussey et al., 2006). Touch screen systems are very versatile, offering flexible means of investigating visual and cognitive function in rodents, and they allow to test behavior between labs in a standardized way (Bussey et al., 1997a,b, 1994).

So far, orienting behavior in rodents has not been studied within a touchscreen-based system. We therefore aimed to develop a task to test for spatial attention and orienting responses in a touch screen system and to validate the sensitivity of the system by inactivating the SC unilaterally in rats. Instead of chronic lesions, pharmacological inhibition, or optogenetic approaches, we used the expression of designer receptors exclusively activated by designer drugs (DREADDs; Burnett and Krashes, 2016), allowing for counterbalanced within-subject comparisons with each animal being its own baseline, without any restrictions in movement after injection of the designer drug clozapine-N-oxide (CNO) or the vehicle.

## Materials and Methods

### Animals and group overview

Adult Long–Evans rats, weighing 250–350 g, were obtained from Charles River Canada. Animals were housed in pairs (except during recovery period after surgery) at a temperature of 21 ± 1°C in a 12/12 h light/dark cycle with lights on at 7 A.M., and food and water available *ad libitum*, except during the weeks encompassing touchscreen training and testing, where the animals were food restricted. All animal procedures were approved by the University of Western Ontario’s Animal Care Committee and followed the guidelines of the Canadian Council on Animal Care. All efforts were made to minimize the number of animals used and any discomfort resulting from surgical or behavioral procedures. Testing occurred during the light part of the light/dark cycle.

A total of 29 rats underwent either sham surgery or SC microinfusion of neuronal-specific inhibitory DREADD viral vectors. After surgical recovery, rats were undisturbed for two to three weeks to allow for sufficient DREADD expression. Following the recovery period, they were each tested in an open-field test (OFT), after systemic injection of either the DREADD activator CNO or vehicle, in a counterbalanced experimental design with a 3-d washout period in between the two tests. After this, rats underwent training in the touch screen task, which took approximately one month. Once animals reached the training criteria, they were tested in the novel orienting task (see below) following either CNO or vehicle. After behavioral testing, animals were perfused and brains were dissected for immunohistochemical verification of DREADD expression.

Four out of 29 rats were tested in the OFT only and euthanized afterwards to generally verify injection coordinates and virus expression. Of the remaining 25 animals, eight animals underwent sham surgery and 17 were injected with DREADDs into the SC. After excluding miss-hits, 14 DREADD animals (seven males, seven females), and eight sham animals (two males, eight females) were tested in both OFT and the touch-screen task, with three additional males tested in OFT only.

### Surgery

Animals were anesthetized with isoflurane, induced with a mixture of 5% isoflurane and 2-l/min oxygen and maintained at 3% isoflurane with 1-l/min oxygen. Meloxicam (1 mg/kg, s.c.) and Baytril (10 mg/kg, i.m.) were administered preemptively. Meloxicam injection was repeated 24 h after the first injection. Animals were secured in a stereotaxic frame and a midline incision was made in the skin on top of the head. A unilateral burr hole (right side) was drilled at the following coordinates from bregma: 1.8 mm medial/laterally, −6.1 caudally, and 4.4 mm ventrally (Paxinos and Watson, 2006); 0.7 μl of the virus solution rAAV5/hSyn-hM4D(Gi)-mCherry (Vector Core of the University of North Carolina at Chapel Hills) was injected at a rate of 0.1 μl/min using a blunt ended 1.0-μl Hamilton syringe (Model 7001 KH SYR, Knurled Hub NDL, 25 gauges, 2.75 in, point style 3; Hamilton). The syringe rested for 1 min before injection and 7 min after injection before slow retraction. In sham animals the needle was introduced, but no injection was made. Silk suture was used to close the wound and rats were given a 21-d recovery period to promote maximal expression of the DREADD protein before testing began. Animals had free access to food and water throughout this waiting period. After OFT testing, animals were food restricted and kept on 90%t of their target body weight to ensure motivation in the operant touch-screen task.

### Behavior: OFT

The open field was a square enclosure of 45.7 × 45.7 cm dimensions with surrounding walls 40.6 cm in height. Animals were administered an intraperitoneal injection of the DREADD ligand CNO (Toronto Research Chemicals, C587520) at a dose of 3 mg/kg in 18% dimethyl sulfoxide (DMSO) in saline, or vehicle (18% DMSO, in saline), 20 min before testing. Animals were then placed in the open-field box and allowed to freely explore it for 20 min while they were tracked using a webcam and ANYmaze software (version 6.33, Stoelting). Default ANYmaze settings did not reliably track Long–Evans rats, in particular the location of their head, because of their non-uniform coat making it difficult to distinguish between the animal’s entire body and the background. In order to enhance tracking, the brightness and contrast of all OFT videos were adjusted by the same degree (reduce brightness, increase contrast), and the ‘Erase Lines’ feature was used, as suggested by ANYmaze technical support, allowing to reliably detect the animal’s head and center of the body (see Movie 1). Total distance traveled, total number of 360° rotations made in either direction (clockwise or anticlockwise), percentage of 360° clockwise rotations, and the cumulative sum of all body and head turn angles throughout the 20-min test period were analyzed. For the cumulative sum of turn angles, clockwise turns were counted as positive and anti-clockwise turns as negative values, so that a positive cumulative sum indicates more clockwise than anti-clockwise turns and vice versa. Turn angle measures have an advantage over complete rotation measures in that they capture the entirety of the animals’ turning behavior, whereas complete rotation measures only count fully completed 360° turns.

Movie 1. ANYmaze video tracking with adjusted settings consistently track the head and body position. Video sample from the OFT, showing ANYmaze tracking using the adjusted settings described in the methods section. The video sample shows consistent tracking of the animal’s head (green) and center (orange) positions, which are used to calculate turning and rotation measures as described in the methods section and supplemental figures. 10.1523/ENEURO.0032-20.2021.video.1

### Behavior: three-choice orienting task

After OFT testing, animals were trained to respond to a three-choice orienting task in the touchscreen testing platform (Bussey Saksida boxes, Campden Instruments Ltd.) to measure orienting behavior and potential signs of visual neglect. The task was adapted from the five-choice serial reaction time task (5CSRTT) commonly used in rodent studies of attention (Bussey et al., 2008; Mar et al., 2013; Fizet et al., 2016), and based on the method of double simultaneous stimulation used to investigate visual selection deficits in human patients (de Haan and Karnath, 2012). The three-choice orienting task used similar basic setup and training protocols as the standard 5CSRTT, but animals were trained to specifically orient toward the center panel. Also, only the leftmost, rightmost and center response panels were used (three-choice) to increase task difficulty and sensitivity for the animals’ orientation during stimulus presentation. Each response panel was a 2.0–3.0 × 2.0–3.0 cm square with a spacing of 5 cm between the side and center panels, and 1.5–2.0 cm away from the grid floor of the box.

#### Pretraining

Initial training consisted of four pretraining stages that acclimated the rats to the testing chamber and taught them the basics of the task such as initiating trials and associating illuminated panel touches with a sugar pellet reward (Mar et al., 2013). A touch is defined as poking the panel with their nose which is detected by the touchscreen system through breakage of the infrared beams along the touch screen panel. The first pretraining stage involved habituating the animals to the testing chamber for 30 min, whereas the remaining pretraining stages progressively taught the rats how to respond to a solid square light stimulus and start a new trial. Rats passed each of these pretraining stage by completing 60 trials in under 60 min.

#### General outline of the task during baseline training and testing sessions

**Figure 1.**
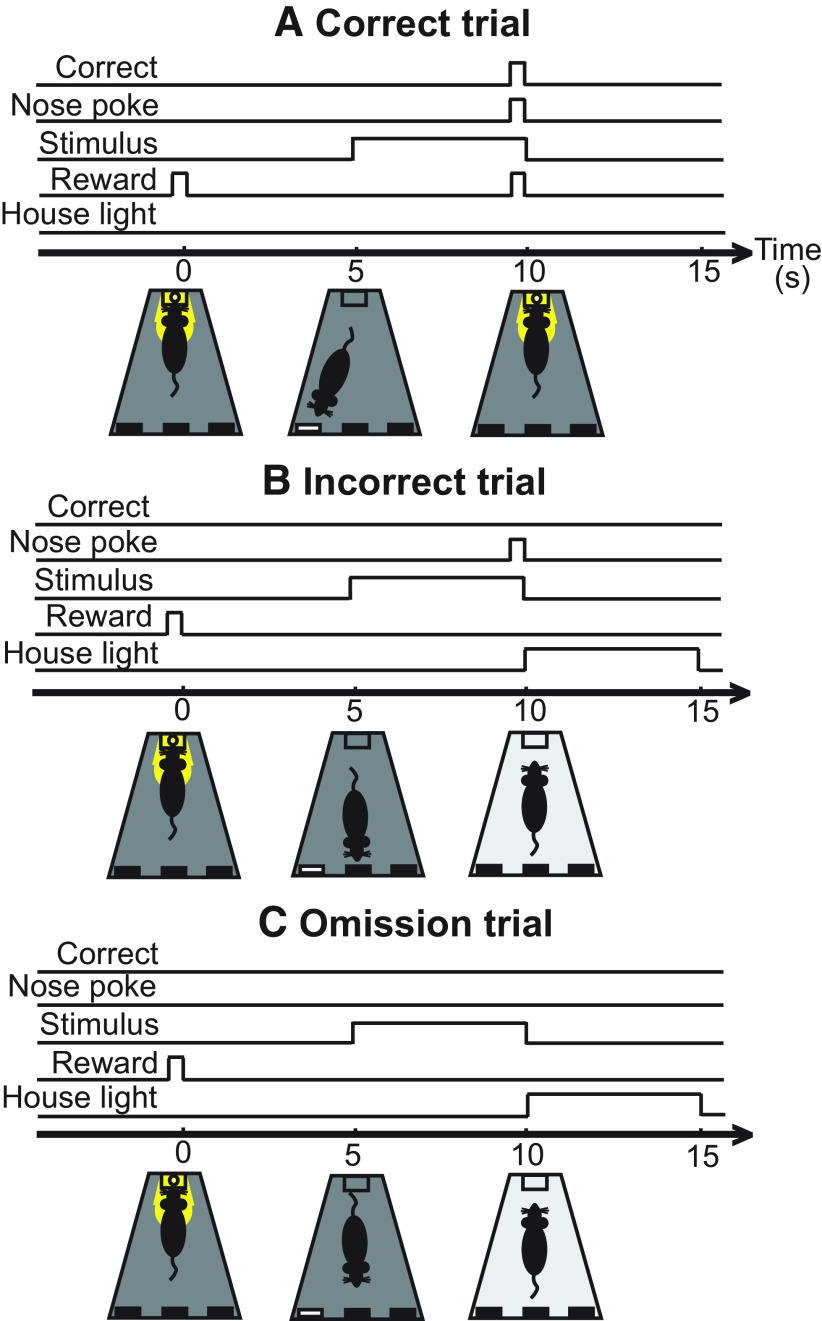
Timeline of events in the touchscreen-based orienting task during baseline training with a difficulty level of 5 s of stimulus presentation. The house light is turned off by default and a sugar pellet (reward) is provided to prime the session, along with illumination of the food magazine. A trial begins as soon as the animal enters the food magazine (0 s), after which the food magazine light is switched off. Then, the stimulus is presented after 5 s, and the animal has to make a response (nose-poke) within a 5-s limited hold of stimulus presentation. ***A***, In a correct trial, the animal has correctly poked the illuminated panel and is rewarded immediately. The house light remains off throughout the trial. ***B***, In an incorrect trial, the animal has poked a non-illuminated panel and receives no reward. The house light turns on immediately after the incorrect response for a period of 5 s to signal a timeout and discourage the animal for an inappropriate behavior. ***C***, In an omission trial, the animal has not poked any panel at all and thus is not rewarded. House light turns on for 5 s right after the limited hold of stimulus presentation (5 s) to signal a timeout. For all trials: the next trial begins after a 5-s ITI with illumination of the food magazine. Note that whenever a sugar pellet (reward) is dispensed, it is accompanied by illumination of the food magazine light, which is then switched off once the animal collects the reward.

House light was turned off by default throughout all training and testing sessions. A session began by delivering a sugar pellet to the food magazine and illumination of the food magazine to prime responding and encourage the animal to initiate the first trial. The first trial started once the animal poked its head into the food magazine (to collect the sugar pellet), followed by a delay interval of 5 s, giving time for the animal to orient toward the screen. At the end of the delay interval, the stimulus was presented for a set “stimulus duration” period, which was varied depending on the stage of training, and this was followed by a “limited hold” period. If the animal correctly nose-poked the panel where the stimulus was presented within the stimulus duration or limited hold, the food magazine was illuminated and a sugar pellet was dropped there. During training sessions, the limited hold was 5 s from the start of stimulus presentation. During testing sessions, the limited hold was removed and the animals could only respond during stimulus presentation, but the stimulus duration was increased to reduce the attentional load of the task. Once the rat collected the reward and exited the food magazine, an intertrial interval (ITI) of 5 s began. After this ITI period, the food magazine light was turned on again and the animal had to poke its head into the food magazine to initiate the next trial. In cases where the animal responded incorrectly by nose-poking a non-illuminated panel, or if the animal omitted a response, a time-out period of 5 s followed that was signaled by turning on the house light. After the time-out period, the house light went back to the normal off state and an ITI of 5 s began. After that, the food magazine light was turned on and the animal could initiate the next trial. Figure 1 outlines the timing of events in the task at a stimulus duration of 5 s for a representative correct (Fig. 1*A*), incorrect (Fig. 1*B*), and omission (Fig. 1*C*) trial.

#### Baseline center attention training

**Figure 2.**
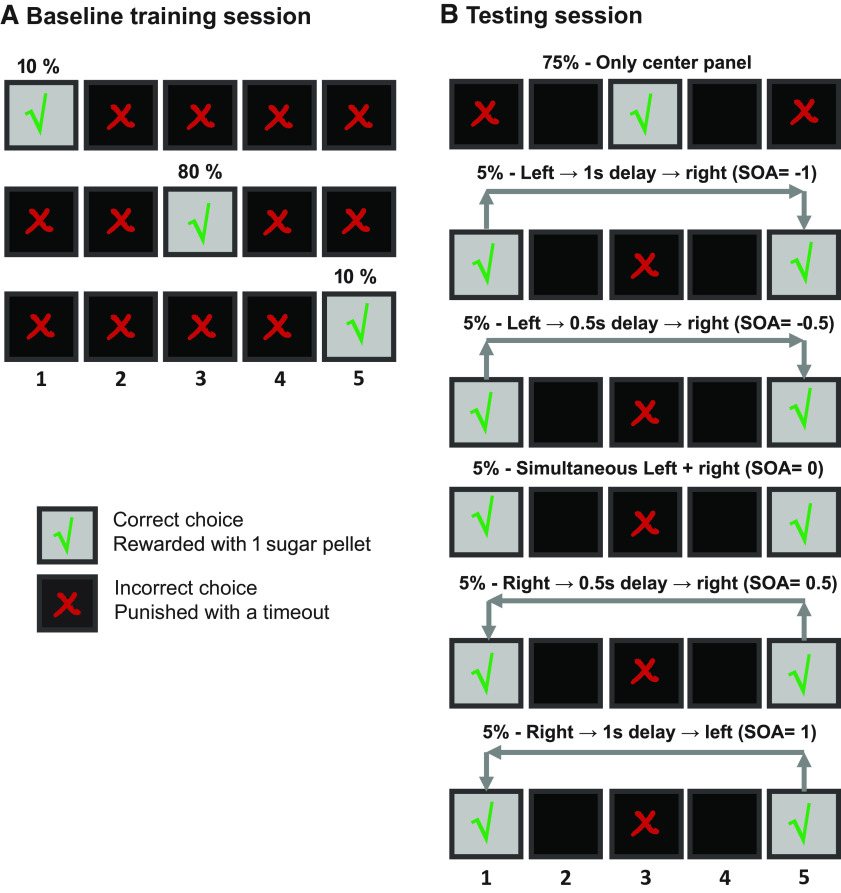
Description of the three-choice orienting protocol. ***A***, After completing all the pretraining steps, the rats undergo baseline attention training, where they learn to expect the center stimulus trial as the most likely trial type, by having the center stimulus appear in 80% of the trials while the left and right stimuli each appear in only 10% of the trials. Animals are rewarded with one sugar pellet on correctly nose-poking the illuminated panel and punished with a timeout and inversion of the house light for poking a non-illuminated panel. The duration of the stimulus presentation is decreased from 30 to 1.5 s through seven stages of baseline training to increase task difficulty. ***B***, Upon passing the criteria, rats perform the testing session after injections of CNO or vehicle. Testing sessions include baseline trials to reinforce the basic strategy the rats must use, in addition to ambiguous trials where flanking stimuli are presented with varying SOAs to test their orienting bias at various difficulties; 75% of the test trials are center-only trials, where only the center stimulus is presented for 5 s. The remaining 25% are double-stimulus trials where the left and right stimuli appear either simultaneously or with a delay (0. 5 or 1 s) with left stimulus preceding the right or vice versa.

After successful completion of pretraining stages, the baseline center attention training commenced. In this stage, rats were trained to expect a center stimulus as the most likely stimulus, and therefore orient toward it more than the left or right response panels. This was done by varying the proportion of trials on which the stimulus was presented at each of the three locations (80% center, 10% right, and 10% left; see Fig. 2*A*). Trials were presented in a randomized order. Baseline training difficulty was incrementally increased by shortening the duration of stimulus presentation. Animals progressed to more difficult stages of baseline training when they reached criteria of >80% total accuracy, <20% omission rate and 100–150 trials completed in 60 min. Stimulus duration decreased from 60 to 1. 5 s during seven baseline stages (60, 30, 20, 10, 5, 2.5, 1.5 s), enforcing enhanced attention as the animal was mastering the task.

#### Test sessions

Once each rat passed the seventh substage of the baseline training (1.5-s stimulus duration), they advanced to the test sessions. In a few cases, performance varied to under 80% accuracy or >20% omission after the initial session where the rats passed training criteria. In this case, the rats were still considered test eligible, especially since the test session was less difficult than training (5-s stimulus duration in testing compared with 1.5 s in training).

In test sessions, the central panel was illuminated on 75% of trials (center-only trials). The remaining 25% of trials were divided equally into five double stimulus trial types: stimuli were presented in both the leftmost and rightmost panels with the following stimulus onset asynchrony (SOA) conditions: simultaneous left + right stimulus presentation; left stimulus followed by right stimulus with either a 0.5- or 1-s SOA; or right stimulus followed by left stimulus with either a 0.5- or 1-s SOA (Fig. 2*B*). Trials were presented in a randomized order. For all double-stimulus trials, a correct response was defined as nose-poking either the left or the right panel after a stimulus was presented, regardless of which one appeared first, and the animal was rewarded accordingly. An incorrect response in double-stimulus trials referred to when the animal nose-poked the center panel (in which no stimulus was presented), or the leftmost or rightmost panels before any stimulus was presented. Touching any other panel between the center and the sides was recorded but neither rewarded nor punished. Trial types were presented in pseudorandomized order. Performance of the full task involved completing 200 trials over 90 min (100 trials in 60 min, for an early cohort). The session was set to end once the animal finished all the trials or once the time limit was reached. Each animal was tested twice with CNO and twice with vehicle, in a counterbalanced order, 20 min following the injection (one early cohort was tested only once for each condition). Tests were performed at least 3 d apart to ensure injection washout, and rats were maintained on center attention baseline training between the test sessions. Data from both sessions following the same injection was combined and used to calculate the respective animal’s performance parameters. The total number of completed trials, accuracy rate, and omission rate were analyzed, as well as the number of responses made to each panel and the respective latencies, separated for each trial type (center only or SOA trials), using the ABET II Touch software (Lafayette Instruments). All training and testing ABET II files are provided with the manuscript as Extended Data 1. Touchscreen response rates were calculated as a percentage of total trials where a response was made (excluding trials where the animals made an omission) to avoid biasing the data with trials in which the animal was not facing the panels (e.g., while grooming, eating), as was the case in a small fraction of total trials. In contrast, omission rate was calculated as a percentage of all trials of a particular trial type presented in a session.

10.1523/ENEURO.0032-20.2021.ed1 Extended Data 1 ABET II Touch software (Lafayette Instruments). Download Extended Data 1, ZIP file.

### Immunohistochemistry

To confirm the expression of the DREADDs in the SC, all animals were perfused with saline followed by 4% paraformaldehyde (PFA), and brains were harvested, postfixed in PFA for 1 h, and stored in 30% sucrose at 4°C until completely sunken. Brains were sliced into four series of 40 μm coronal sections using a freezing microtome (Microm HM 560 M) and stored at −20°C in cryoprotectant solution. One series of the sections was used for immunohistochemistry. Free floating tissue sections were thoroughly washed in 0.1 m PBS between incubations and all incubations were performed at room temperature with gentle agitation. Sections were blocked with 1% H_2_O_2_ in 0.1 m PBS for 10 min and preabsorbed in PBS+ (0.1% bovine serum albumin, 0.4% Triton X-100 in PBS) followed by overnight incubation with rabbit anti mCherry (Abcam catalog #ab167453, RRID: AB_2571870) in PBS+. Subsequently, the sections were incubated with biotinylated goat anti-rabbit (Vector Labs catalog #BA-1000, RRID: AB_2313606) for 1 h (1:500 in PBS) and by the avidin horseradish peroxidase for 1 h (ABC-elite, 1:1000 in PBS; Vector Laboratories). Finally, the peroxidase complex was visualized by exposure for 10 min to a chromogen solution containing 0.02% 3,3’-diaminobenzidine tetrahydrochloride. At the end of the staining protocol, sections were washed thoroughly with 0.1 m PB, mounted onto plus-charged glass slides with 0.3% gelatin in distilled water and cover-slipped with DPX mounting medium (EMD Millipore). Imaging was performed using a Nikon Eclipse Ni-U upright microscope with a DS-Qi2 high-definition color camera and imaging software NIS Elements Color Camera (Nikon Instruments).

### Statistical analysis

All statistical analyses were conducted using SPSS (IBM).

#### Testing for normality, outliers, homogeneity of variance, and sex

Before conducting statistical analyses, data were scanned for normality using the Shapiro–Wilk test, for outliers using box and whisker plots, and for homogeneity of variance assumptions for relevant analyses. This was conducted for each measure of interest in the OFT and touch screen three-choice orienting task. For data that did not exhibit a normal distribution according to the Shapiro–Wilk test (*p* < 0.05), we conducted nonparametric Mann–Whitney or Friedman tests, instead of independent *t* tests and one-way repeated measures ANOVA (see below). For data that did not violate normality but violated the assumption of homogeneity of variance, an adjusted *p* value for *t* tests was used. Data of both sexes were merged throughout the study, as there were no statistically significant differences between sexes, except for overall locomotor activity. The introduction of difference scores for each animal between CNO and vehicle trials normalized the data and eliminated the difference in overall locomotion.

#### OFT

A CNO-vehicle difference score was calculated for total distance traveled, total rotations, the percentage of clockwise rotations, the head turn angle sum and the turn angle sum. Independent *t* tests were conducted for all measured except total distance traveled, comparing the DREADD group with the sham group, and *p* values were chosen accordingly based on whether the assumption of homogeneity of variance was met (only case is for turn angle sum). For the total distance, a Mann–Whitney *U* test was performed.

#### Touch screen baseline performance

For training data across days on the three-choice orienting task, pretest baseline training days just before respective CNO or vehicle testing days were analyzed, using only animals that had two CNO and two vehicle test days (four pretest days in total). Accuracy percentage, omission percentage, reward collection latency, and correct response latency were analyzed. After determining no difference in performance across pretest days between DREADD and sham animals, data of the two groups were merged.

Accuracy percentage, omission percentage and correct response latency were analyzed separately for left stimulus trials, center stimulus trials, and right stimulus trials. Preliminary analysis using repeated measures ANOVA or Friedman test revealed no significant difference between pretest days, after adjusting for multiple comparisons, in percentage accuracy, percentage omissions or correct response latency, for any of the three trial types. Therefore, data from all four pretest days were combined and used to calculate overall trial-type-specific percentage accuracy, percentage omission and correct response latency. Then, the percentage accuracy, percentage omission and correct response latency were compared between trial types using a one-way repeated measures ANOVA or Friedman test, depending on whether normality and homogeneity of variance criteria were met.

#### Touch screen testing performance

For center stimulus trials on the three-choice orienting task testing sessions, the CNO-vehicle difference score was calculated for the percentage left responses, center responses, right responses, and omissions for each animal. Additionally, we calculated the CNO-vehicle difference score for the center response latency. Independent *t* tests or Mann–Whitney *U* tests were conducted for each of these measures comparing the DREADD group with the sham group.

Double stimulus trials were analyzed as described in Johnston et al. (2016). The proportion of responses to the ipsiversive choice, the rightmost panel, were computed and plotted as a function of SOA. Values were fitted with a logistic function and plotted for each individual animal, as well as for each group using the average value of all animals in that group for each SOA. The midpoint of this psychometric function represents the point of equal selection (PES), which is the SOA value at which the proportion of rightward responses equals the proportion of leftward responses (0.5 proportion of right responses).

Curves were fitted and PES values were calculated only for animals that had more than five trials on all SOAs in which they made a response, which led to the exclusion of one sham animal and five DREADD animals. CNO effects were so strong on some DREADD animals that they responded almost always to the right, so the PES could not be calculated. In these cases, the smallest SOA value used to generate the fitted curve (SOA = −1) was used as a conservative estimate. A two-way repeated measures ANOVA was performed with group (sham vs DREADD) as a between subject factor and injection (vehicle vs CNO) as a within subject factor.

## Results

This study examined the effects of DREADD-induced transient deactivation of the right SC on orienting behavior in Long–Evans rats. The OFT was initially used to determine changes in spontaneous turning and rotation behavior on deactivating the right SC, followed by a novel touchscreen-based three-choice orienting task to detect any changes in spatial attention and orienting behavior. At the end of data acquisition, immunohistochemistry was performed postmortem to confirm DREADD expression.

### DREADD expression

**Figure 3.**
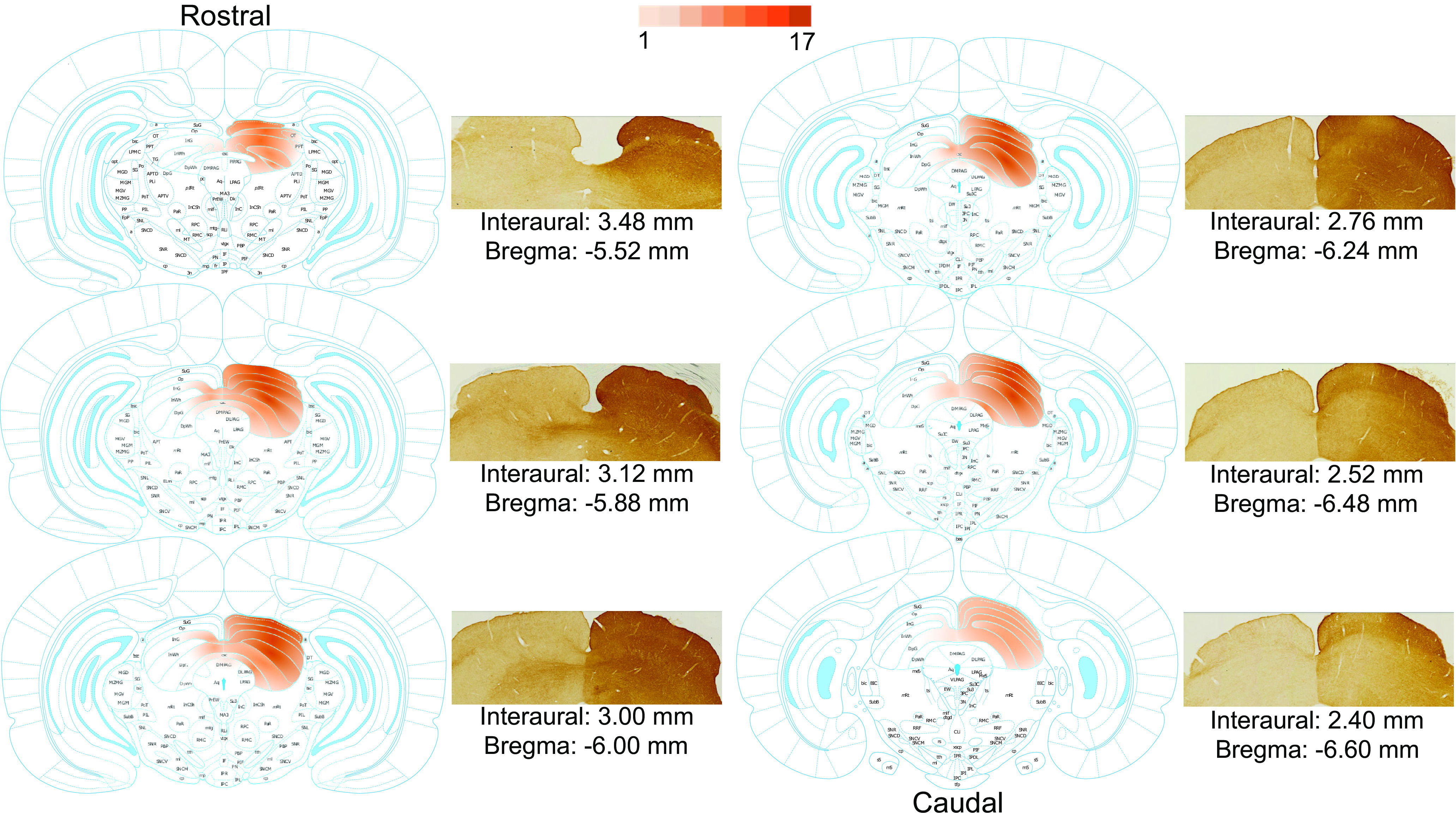
DREADD expression in the SC across all DREADD-expressing animals. Expression is displayed across six slices, from bregma −5.52 mm as the most rostral, to bregma −6.60 mm as the most caudal slice. The color gradient on the SC represents the number of animals that had DREADD expression at each subregion of the SC: the darker the gradient at a subregion, the higher the number of animals that had DREADD expression in that subregion. Each schematic slice is accompanied by an immunohistochemistry photograph of the same slice from a representative animal. The images of the slices were taken from *The Rat Brain Atlas in Stereotaxic Coordinates* from Paxinos and Watson (2006).

All animals underwent postmortem immunohistochemistry to identify the regions with DREADDs expression. No expression was observed in the sham operated animals. In four animals, the injection was identified as missed-hit as no expression of the virus was observed in their right SC. These animals were excluded from the study. In all the remaining animals, DREADDs were uniformly expressed throughout a large portion of the right SC, from superficial layers down to the deeper layers and from rostral to caudal sections (Fig. 3 for a representative example). Figure 3*A*, darker colors, demonstrates areas with stronger expression of DREADDs. In some cases, DREADD expression included fibers crossing the midline and invading the left SC, evident by fiber-shaped staining in the region of the commissure, or a very low level of expression in deeper layers of the contralateral SC as well the periaqueductal gray (PAG) area inferior to the SC.

### Behavior: OFT

The OFT was implemented to evaluate spontaneous orienting during exploratory locomotor behavior. Ipsiversive (rightward or clockwise) body turns and head turns were analyzed. Head turns were measured through the head turn angle and full 360° rotations, whereas body turns were measured through the turn angle sum. Differences between behavior after CNO and vehicle injections (CNO-vehicle scores) were calculated for each animal and averaged for each group (sham vs DREADD-expressing animals).

**Figure 4.**
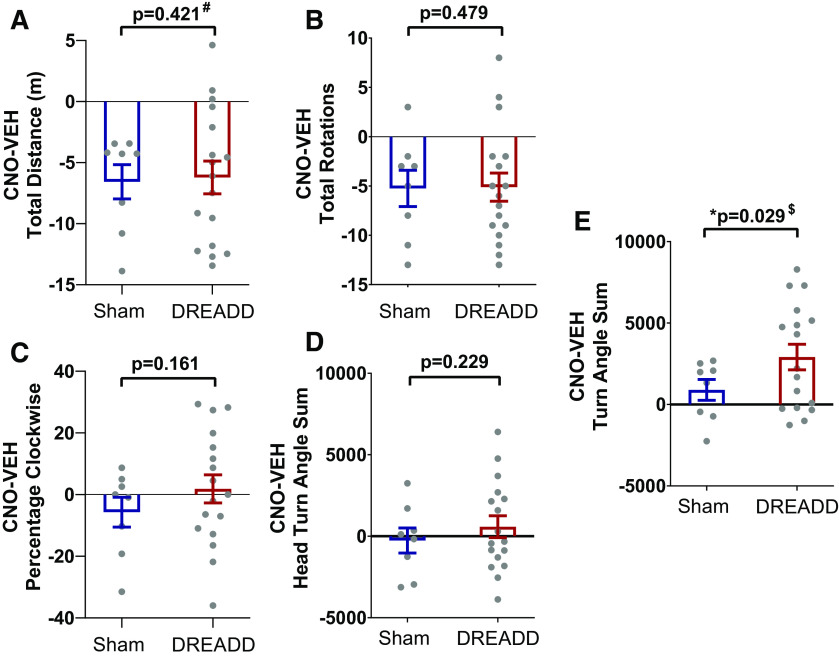
Body turning but not head turning behavior is increased following CNO in DREADD but not sham animals. Rats spontaneously explored an enclosed arena 20 min after injection of vehicle or CNO. A difference score was calculated for each measure of interest between CNO and vehicle for each animal, and the sham and DREADD groups were compared using independent *t* tests or Mann–Whitney *U* test (#). ***A***, There was no difference in the CNO-vehicle score of total distance traveled. ***B***, No difference in total 360° rotations. ***C***, No difference in percentage clockwise 360° rotations. ***D***, No difference in the sum of all head turn angles made during the 20-min test (clockwise head turns were denoted as positive and anticlockwise head turns as negative). ***E***, There was a significant increase in the CNO-vehicle score of the turn angle sum (clockwise body turns were denoted as positive and anticlockwise body turns as negative). All values shown are mean ± SEM, with individual dots representing individual animals; * significant effect with *p* < 0.05, $ denotes adjusted *p* value based on lack of homogeneity of variance. For more information on how turn angles were measured, see Extended Data Figure 4-1.

We found no significant difference between sham and DREADD animals in CNO-vehicle scores in locomotor activity measures. These included the total distance traveled (*U* = 72, *z* = 0.233, *p* = 0.421; Fig. 4*A*) and the total number of 360° rotations (*t*_(23)_ = 0.054, *p* = 0.479; Fig. 4*B*). However, CNO reduced overall locomotor activity in both groups of animals, as indicated by a negative CNO-vehicle score for total distance and total rotations made throughout the 20-min test. In terms of head turning behavior, CNO-vehicle scores of percentage clockwise rotations (*t*_(23)_ = 1.104, *p* = 0.161; Fig. 4*C*) and head turn angle sum (*t*_(23)_ = 0.758, *p* = 0.229; Fig. 4*D*) were not significantly different between sham and DREADD animals (for more information on turn measurements, see also Extended Data Fig. 4-1). However, DREADD animals showed a significantly higher CNO-vehicle score for turn angle sum (*t*_(22.04)_ = 1.991, *p* = 0.029; Fig. 4*E*). Taken together, these results indicate that unilateral DREADD-induced inactivation of the SC increased ipsiversive whole body turning as measured by the turn angle sum. In contrast, neither head turning behavior, as measured by percentage clockwise rotations of the head and head turn angle sum, nor the number of total rotations were significantly changed by SC inhibition in these animals.

10.1523/ENEURO.0032-20.2021.f4-1 Extended Data Figure 4-1 Turn measure examples. Visual depiction of turn angle measures recorded by ANYmaze software. ***A***, Turn angle: a vector of movement from one position of the animal’s center point to the next is created. For each vector, the angle between it and the previous vector is calculated with anti-clockwise movement being negative and clockwise movement being positive (ANYmaze). ***B***, Head turn angle: for each position of the animal’s head, a vector is created from the animal’s center point to the head. The angle between this vector and the same vector for the previous position of the animal’s head is calculated, with anti-clockwise movement being negative and clockwise movement being positive. ***C***, Example calculation from successive head turns: panels are numbered from left to right as C1, C2, C3, C4. C1 to C2 head turn angle = +75; C2 to C3 head turn angle = +45; C3 to C4 head turn angle = –35. Turn angle sum is the sum of all turn angles, measured as in panels ***A***, ***B***, in a particular time period (e.g., the head turn angle sum for time period C1–C4 = 75 + 45 – 35 = +85). To complete a full rotation, an animal must make consecutive turns that add up (cumulative turn angle) to 360°. Reversal (turning towards the opposite direction) at any point before the cumulative angle reaches 360° resets the cumulative angle to 0 and starts adding up successive turns to the opposite direction (e.g., considering panels C1–C4, the cumulative angle counted towards a full rotation is –35 because the animal made a reversal, despite the head turn angle sum being +85). Because of the effects of the reversal, we believe the turn angle sum is a more accurate representation of turning behavior than complete 360° rotations. Download Figure 4-1, EPS file.

### Behavior: three-choice orienting task training

Almost all rats progressed quickly through the pretraining and baseline training sessions described in the methods section and Figures 1, 2. On average, rats took ∼2 d to reach passing criteria on each of the pretraining and baseline training stages, except the most difficult training stage which on average took 3 d to learn.

**Figure 5.**
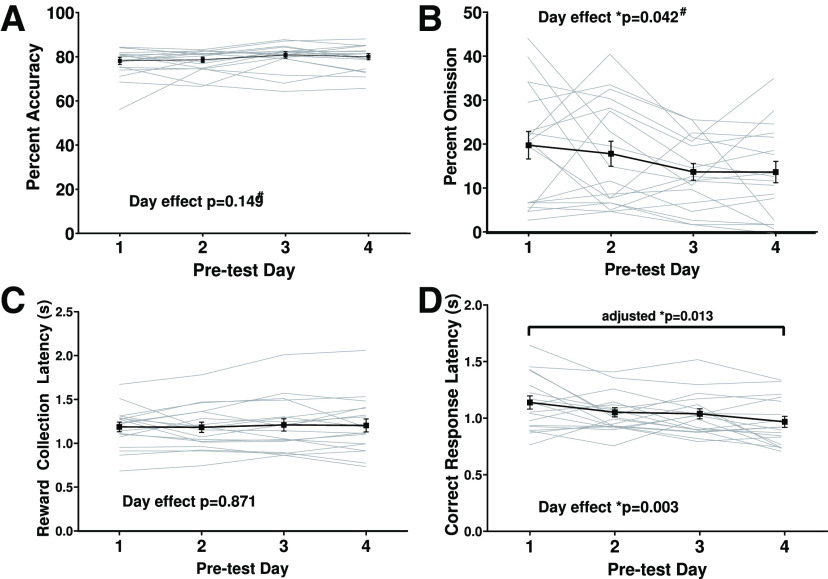
Performance and motivation are stable across pretest days, but animals omit fewer trials and respond more quickly by the last pretest day. Animals underwent baseline training and were then tested up to four times. These graphs show performance parameters on the days before testing days for both sham and DREADD animals combined, which was analyzed using repeated measures ANOVA or nonparametric Friedman test (#). ***A***, Overall accuracy did not change across pretest days. ***B***, Percentage omission significantly decreased across pretest days. ***C***, Reward collection latency was unchanged across pretest days, indicating stable performance and motivation across pretest days. ***D***, Correct response latency significantly decreased across pretest days, adjusted *p* refers to comparison between day 1 and day 4, which was adjusted for multiple comparisons. These results indicate that by undergoing test sessions which include more reward and less punishment, animals learned to omit fewer trials and respond more quickly. All values shown are mean ± SEM, with light gray lines representing individual animals; * significant effect with *p* < 0.05. For more information on progression through all training stages, see Extended Data Figure 5-1.

The purpose of baseline center training sessions was to train the rats to orient toward the center stimulus more than the flanking stimuli by using a higher proportion of center stimulus trials (80% center, 10% left, 10% right). Given that the experimental design involves repeated testing of animals, it was important that animals maintained a stable baseline performance between test sessions, especially on pretest days before their CNO or vehicle test sessions. The baseline training was able to achieve this stable performance, as demonstrated by the lack of significant change across four pretest days in total accuracy (χ^2^(3) = 5.333, *p* =0.149; Fig. 5*A*), indicating overall stable performance, and reward collection latency (*F*_(3,51)_ = 0.236, *p* = 0.871; Fig. 5*C*), indicating no change in motivation. Interestingly, both omissions (χ^2^(3) = 8.186, *p* = 0.042; Fig. 5*B*) and average correct response latency (*F*_(3,51)_ = 5.257, *p* = 0.003; *p* = 0.013 for comparison between day 1 and day 4 adjusted for multiple comparisons; Fig. 5*D*) decreased over pretest days, likely because of the interim test sessions between these pretest days containing more rewarded trials and a smaller chance of punishment. For information on progression through training stages, see also Extended Data Figure 5-1.

10.1523/ENEURO.0032-20.2021.f5-1 Extended Data Figure 5-1 Time in training stages. Rats progress quickly through all training stages in the five-choice orienting task. Pretraining stages (habituation to must initiate) acclimate rats to the chambers and them in a stepwise manner to associate nose-poking stimuli on the screen with reward. All rats passed quickly through pretraining stages, taking at most 4 d to reach criterion and an average of about 5 d to finish all of pretraining. Baseline training stages involve training the rats to orient towards the center panel, with a stimulus being shown 10% in the left panel, 80% in the center panel, and 10% in the right panel. To pass, rats must achieve an overall accuracy of 80% with fewer than 20% omitted trials. Rats progressed quickly through baseline 1 (60-s stimulus duration) to baseline 4 (10-s stimulus duration). Even on the most difficult stage, rats took an average of 3 d to reach criterion and just under 12 d to complete all baseline stages. Download Figure 5-1, EPS file.

**Figure 6.**
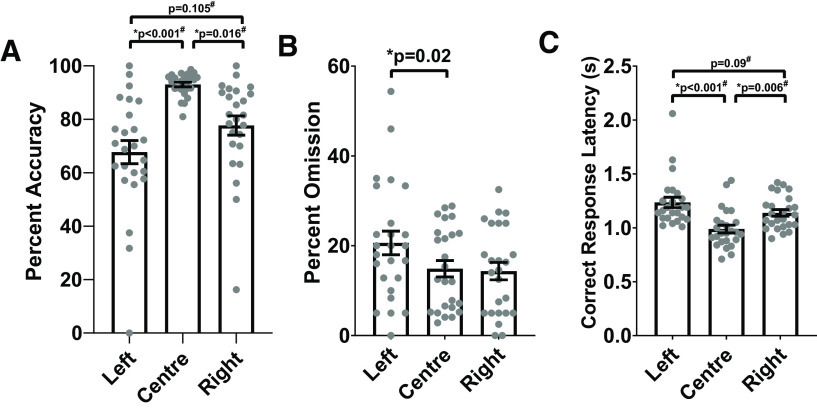
Animals show strong center bias on pretest days. Data from all pretest days were combined to analyze trial-type-specific performance using repeated measures ANOVA or nonparametric Friedman test (#). All *p* values shown are adjusted for multiple comparisons. ***A***, Animals were more accurate in response to center trials. ***B***, The omission rate was higher to left side trials than to center trials. ***C***, Correct responses to center trials were faster than to side trials. All values shown are mean ± SEM, with individual dots representing individual animals; * significant effect with *p* < 0.05.

In order to investigate the orienting bias produced by baseline training in more detail, we analyzed trial-type-specific responses. For this purpose, all trials from pretest days were combined after confirming that trial-type-specific measures of interest did not significantly differ between the different pretest days (adjusted for multiple comparisons). Trial-type-specific responses during pretest days showed a strong center bias, with higher accuracy on center trials compared with left or right trials (main trial type effect: χ^2^(2) = 24.437, *p* < 0.001; multiple comparisons with adjusted *p* values shown on Fig. 6*A*) and a shorter correct response latency on center trials compared with left or right trials (main trial type effect: χ^2^(2) = 24.089, *p* < 0.001; multiple comparisons with adjusted *p* values shown on Fig. 6*C*). Interestingly, while omission percentage on left trials was significantly higher than center trials, omission percentage on right trials was comparable to center trials (main trial type effect: *F*_(2,42)_ = 5.569, *p* = 0.007; comparison between left and right trial types adjusted for multiple comparisons shown on Fig. 6*B*). Overall, the three-choice orienting task training successfully produced a strong center bias, as also easily observed in a representative video taken during one of the baseline training days on stage 7 (see Movie 2).

Movie 2. Baseline center training leads to a strong center bias. Video sample from the touch screen orienting task baseline training. The video sample shows the animal orienting towards the center before the stimulus is shown and correctly responding to the center stimulus and receiving a reward (00:00–00:02 for orienting before a stimulus is shown, 00:02–00:04 for responding to the stimulus). A similar sequence of events can also be seen in the following three trials (00:12–00:15, 00:28–00:31, 00:40–00:44). Following a correct response, the animal collects its reward and initiates a new trial. The last trial in the video shows an incorrect side trial, where the stimulus was shown on the side, but the animal’s center bias made it incorrectly choose the center (00:51–00:54). 10.1523/ENEURO.0032-20.2021.video.2

### Behavior: three-choice orienting task testing

The testing protocol was comprised of 75% baseline center trials to retain the animals’ center bias, and 25% double stimulus side trials with varying SOAs to test the orienting preference to varying degrees following CNO or vehicle injections (Fig. 2*B*). Separate analyses were conducted for baseline center-only trials and double-stimulus trials.

#### Baseline center-only trials

**Figure 7.**
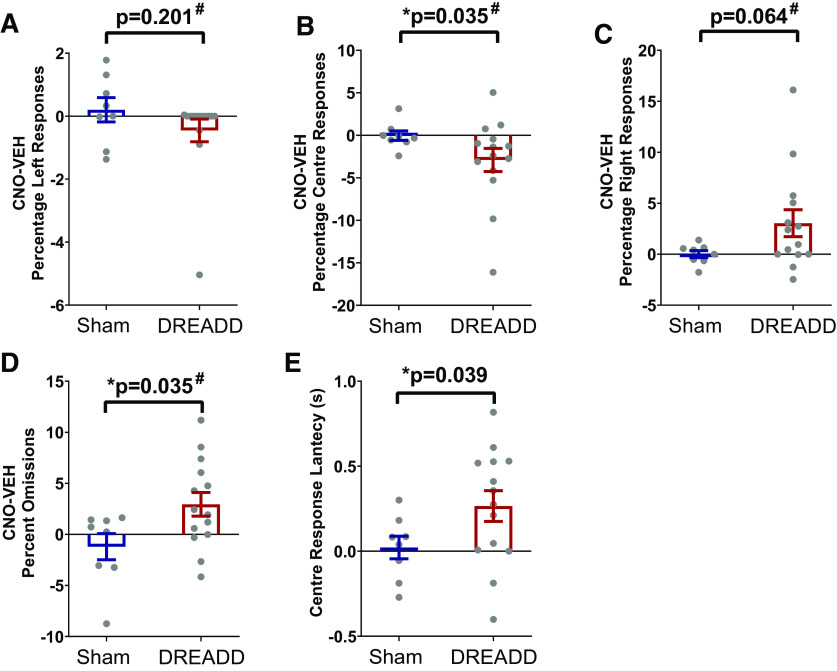
CNO reduces center bias in DREADD but not sham animals during testing. Test sessions included 75% center trials, similar to the ones shown during baseline training. A CNO-vehicle score was calculated for each parameter of interest. Depending on the distribution, an independent *t* test or Mann–Whitney *U* tests (#) were conducted to compare sham and DREADD animals. ***A***, Left Responses were unchanged. ***B***, There was a significant decrease in the percentage of correct center responses and (***C***) a trend to an increase in rightward responses. ***D***, There was also a significant increase in the percentage of omissions in DREADD animals. ***E***, A reduction in center bias can also be seen by an increase in the time it took DREADD animals to respond correctly to the center following CNO. All values shown are mean ± SEM, with individual dots representing individual animals; * significant effect with *p* < 0.05.

Response choice indicates whether the animal made a left, center or right response, although only center choices were rewarded, and the side responses were punished. As in previous analysis, CNO-vehicle difference scores were computed for the percentage of left responses, center responses, right responses and omissions. In addition, the difference for latency to respond correctly to the center was calculated. There was a significant difference in CNO-vehicle scores between sham and DREADD animals for the percentage of center responses (*U* = 83, *z* = 1.834, *p* = 0.035; Fig. 7*B*), percentage omissions (*U* = 29, z = −1.834, *p* = 0.035; Fig. 7*D*), and center correct response latency (*t*_(20)_ =1.865 *p* = 0.039; Fig. 7*E*). Specifically, DREADD animals, but not sham animals, showed lower percentage center responses and higher scores in percentage omissions and correct response latency after CNO injections compared with vehicle controls. There was also a trend for an increase in CNO-vehicle scores of percentage right responses (*U* = 33, z = −1.572, *p* = 0.064; Fig. 7*C*), but no effect on the percentage of left responses (*U* = 69, *z* = 0.948, *p* = 0.201; Fig. 7*A*). Overall, this shows that CNO slightly reduced the center bias in DREADD but not sham animals, causing them to respond less accurately, more slowly, and with a higher chance of omissions when faced with a center trial.

#### Double-stimulus trials with varying SOAs

The goal of double-stimulus trials was to assess the extent of rightward bias produced by unilateral SC inactivation. On the psychometric function, a rightward bias would manifest as a leftward shift and smaller PES values. A weak rightward bias would show a left shift at negative SOAs only, whereas a strong rightward bias would show a left shift at all SOA values.

**Figure 8.**
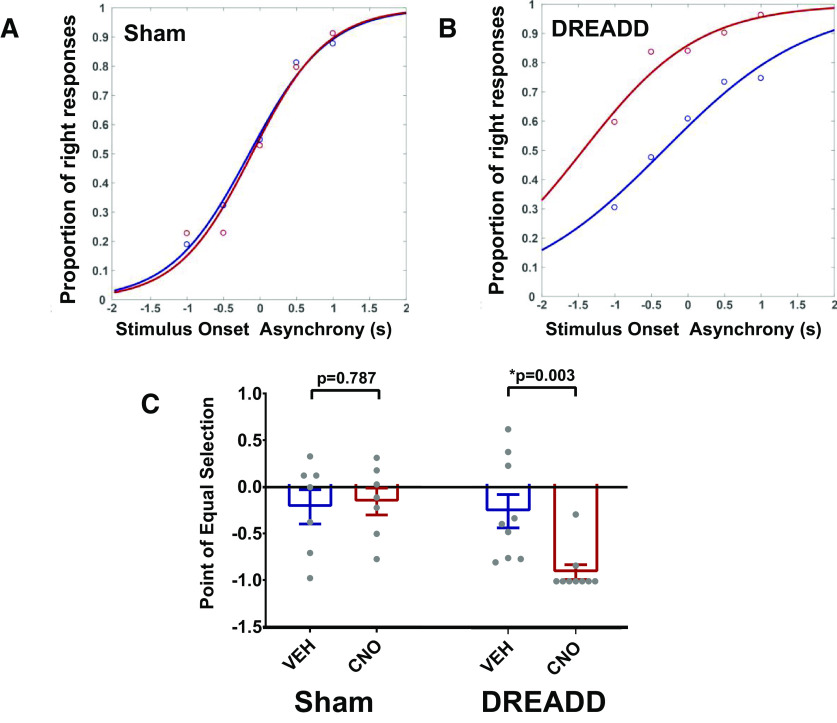
CNO produces a strong rightward bias in DREADD animals. ***A***, ***B***, During test sessions, SOA trials were used to assess the extent of rightward bias produced by CNO in DREADD animals. Circles in ***A***, ***B*** indicate the average proportion of right responses at that particular SOA for the entire group with either vehicle (blue) or CNO (red). A logistic function was fitted to the proportion of rightward responses for each animal, as well as for the whole group. DREADD animals, but not sham animals, showed a left shift of their response profile after CNO injection. ***C***, The PES (0.5 right responses) was extrapolated from each individual animal’s curve following either vehicle or CNO injection. A value of −1 was used as a conservative estimate of PES for animals where the logistic function revealed an extreme rightward bias that could not be extrapolated to a PES. A two-way ANOVA was conducted to compare the PES within each group, which showed a significant decrease in PES in the DREADD group, but no change in the sham group. Values are mean ± SEM, with individual dots representing individual animals; * significant effect with *p* < 0.05. For more information of each single animal’s performance, see Extended Data Figure 8-1 for sham animals and Extended Data Figure 8-2 for DREADD animals.

In the sham group, CNO had no effect on the proportion of right responses, as can be seen by the curves generated from the average of all sham animals at each SOA (Fig. 8*A*; for individual animals, see Extended Data Fig. 8-1). In contrast, CNO injection in DREADD-expressing animals shifted the fitted curve for the proportion of right responses significantly to the left (Fig. 8*B*; for individual animals, see Extended Data Fig. 8-2), indicating a strong rightward bias. A two-way ANOVA conducted with respective PES values supported this rightward bias (Fig. 8*C*,*D*). There was a significant interaction between group and injection (*F*_(1,4)_ = 6.422, *p* = 0.024). *Post hoc* Bonferroni tests adjusted for multiple comparisons revealed a significant decrease in DREADD PES following CNO compared with vehicle (*p* = 0.003), and no change in sham PES following CNO compared with vehicle (*p* = 0.787).

10.1523/ENEURO.0032-20.2021.f8-1 Extended Data Figure 8-1 No rightward bias in individual sham animals. Sham animals do not show a rightward bias in double stimulus trials following CNO injection. Individual psychometric curves for sham animals showing their performance on double stimulus trials within the SOA range –1 to +1 following either vehicle (blue) or CNO (red) injections. A rightward bias would manifest as a leftward shift in the psychometric curve, indicating a higher proportion of rightward responses. As can be seen by the individual curves, sham animals did not exhibit a rightward bias following CNO injection compared to vehicle. Download Figure 8-1, EPS file.

10.1523/ENEURO.0032-20.2021.f8-2 Extended Data Figure 8-2 Rightwards bias in individual DREADD animals. DREADD animals show a rightward bias in double stimulus trials following CNO injection. Individual psychometric curves for DREADD animals showing their performance on double stimulus trials within the SOA range –1 to +1 following either vehicle (blue) or CNO (red) injections. Animals are numbered left to right, top to bottom (row 1: animals 1–4; row 2: animals 5–8, row 3: animal 9). A rightward bias would manifest as a leftward shift in the psychometric curve, indicating a higher proportion of rightward responses. Several rats showed extremely strong rightward bias, as indicated by almost exclusive rightward responses regardless of SOA (animals 3, 5, 8, and 9). Other animals showed a more modest rightward bias (animals 2, 4, and 6) and only two rats showed no rightward bias following CNO (animals 1 and 7) Download Figure 8-2, EPS file.

## Discussion

In this present study, we developed a touchscreen-based experimental approach to measure spatial attention and orienting responses in rodents. Given the well-established role of the SC in orienting behavior of rodents, we validated the novel task through unilateral SC inactivation using DREADDs. Our results show that the touchscreen three-choice orienting task was highly sensitive to DREADD-induced SC inactivation, providing a variety of measures to assess orienting bias.

### DREADD expression

In most of the animals, DREADDs were expressed throughout a large portion of the right SC, from superficial layers down to the deeper layers (Fig. 3). In some animals, we also observed a low level of expression in deeper layers of the left SC. However, the strong imbalance of expression between the left and right SC would still be expected to result in behavioral changes associated with the stronger deactivation of the right side, as supported by our behavioral results. Minor expression was also observed in PAG area in some animals. In a study by Geula and Asdourian (1984), muscimol injections were made into the PAG area in addition to the SC, to observe the impact on animal’s circling and bodily asymmetry. They only observed significant measures of bodily asymmetry and no significant effect on circling (Geula and Asdourian, 1984). Since we did not observe any bodily asymmetry, the observed orienting changes in the present study are likely associated with the strong DREADDs expression in the right SC.

### Open-field analysis

We first analyzed OFT behavior to detect general alterations in the animals’ spontaneous locomotor behavior after deactivation of the right SC. We found that CNO increased clockwise body turning behavior, but not head turning behavior or the percentage of 360° clockwise rotations in DREADD animals. The observation of a higher ipsiversive body turn angle is consistent with a deficit in orienting to the contralateral visual field as a consequence of unilateral SC deactivation and the ipsilesional circling behavior that has previously been reported in lesion studies of the SC (Kirvel et al., 1974; Di Chiara et al., 1982; Geula and Asdourian, 1984; Northmore et al., 1988; Tehovnik, 1989). Our experimental approach also provides a method of optimizing video tracking for Long–Evans rats to aid in the reliable automated detection of the head, which is more difficult for these hooded rats compared with other strains with a uniform coat color. Our findings clearly demonstrate the importance of accurately defining and quantifying measures of turn and rotations, as inhibition of the SC in this study did not induce changes in turning and rotation measures that only use the orientation of the animals’ heads.

### SC manipulations impact orienting responses

Various studies reported that damage to the SC impaired the ability to perform orienting movements toward visual stimuli presented in the hemifield contralateral to the lesioned SC, particularly those presented in the periphery (Goodale and Murison,1975; Schneider, 1975; Dean and Redgrave, 1984). The balance of neuronal activity between the two SCs has been proposed to play a role in selection and initiation of the direction of the motor output (Carello and Krauzlis, 2004; Horwitz, 2004). Both of these task-related processes are important for performance on our three-choice orienting task, as well as for other tasks typically analyzed after SC inactivation. Accordingly, unilateral optogenetic stimulation of the SC revealed that the excitation of SC neurons resulted in contraversive movements, while their inhibition caused an ipsiversive bias (Stubblefield et al., 2013). Pharmacological studies have also consistently supported these findings: Dean et al. (1988) examined the effect of unilateral excitation of SC neurons in rats using pharmacological manipulations and reported contraversive head movements. Other studies reported postural asymmetry following unilateral pharmacological deactivation of the SC in rats (Imperato and Di Chiara, 1981; Geula and Asdourian, 1984). We did not observe any noticeable changes in the rats’ posture on CNO administration in any of our experiments. However, studies reporting bodily asymmetry often used the GABA agonist muscimol for SC inhibition, which might be responsible for postural asymmetries observed, since manipulation of GABAergic mechanisms has previously been reported to cause postural asymmetry and muscular rigidity in rats (Turski et al., 1984). The DREADDS used in this study affected all cell types. Indeed, more recent studies that applied optogenetic techniques to unilaterally deactivate the SC did also not report on any postural asymmetry (Stubblefield et al., 2013; Kopec et al., 2015). Taken together, our findings were consistent with previous literature demonstrating deficits in contraversive orienting following unilateral SC lesions or unilateral pharmacological or optogenetic manipulations of SC activity. Compared with optogenetics, DREADDs provided a less restrictive technique for exploring orienting behavior, as there was no need to tether the animal’s head which may limit the range of their orienting behavior.

### The importance of a center bias

Training on the novel three-choice orienting task was successful in developing a center-orienting preference, evident from higher accuracy and shorter response latency on center trials compared with left or right trials during the test sessions. Accuracy and reward collection latency were consistent across all pretest days, indicating that animals returned to approximately the same baseline orienting bias and motivation before each test session. In the varying SOA trials during test sessions, DREADD but not sham rats showed a substantial left shift in their rightward response psychometric function following CNO injection. This indicated a rightward bias which was also shown by a significant decrease in PES in DREADD animals post-CNO compared with vehicle. The rightward bias was rather extreme in some cases, causing animals to respond to the right on all trials in all SOAs, even when they were primed to respond toward the left side in −0.5 and −1 SOA trials, indicating an ipsiversive orienting bias and contraversive neglect after CNO administration. Even in analysis looking at baseline center-only trials, a rightward orienting bias following CNO could be detected during test sessions. Animals were still very accurate on these center-only trials (center response rate of >90%), but there was a significant decrease in CNO-vehicle score of percentage center responses, whereby animals chose the center choice less frequently and tended toward choosing the right choice more frequently instead, although only the center choice was illuminated and choosing the non-illuminated right panel was punished. Similar findings were reported by Sinnamon and Garcia (1988) using an operant orienting task with two lateral choices and one center choice; however, their results also showed a neglect to center positions after unilateral lesions of the SC, probably because of a lack of enforcing and maintaining a center orientation bias. Without this bias, it is difficult to interpret the behavioral data, as the animal might simply shift its body posture and attention toward the preferred side.

### Benefit of the touchscreen system

The touchscreen-based orienting task is a purely visual method that provided great advantages in terms of precise control of the stimulus duration and location. Compared with tasks that require animals to exert pressure to push a door or a lever, our orienting task benefits from detecting the nose-poke via infrared beam without requiring any pressure, providing a more direct association with orienting behavior (Bussey et al., 2008). Cook et al. (2004) found that rats learned a visual discrimination much faster when they were required to nose-poke the stimuli on a touchscreen as opposed to pushing a lever underneath the stimuli, likely because of differences in the spatial contiguity of stimuli and responses (Cook et al., 2004). Spatial contiguity between stimulus and response is crucial when orienting toward the stimulus is the main goal of the task, and it allows stimulus location to be varied without interfering with the nature of the task or learning speed. The novel task presented here can be easily adapted to studies of orienting response to various directions, since stimuli can be presented anywhere on the touchscreen, or response to stimuli of varying shapes, sizes and salience (brightness). Based on our findings, training rats on this orienting task can be achieved in a relatively short period of time, reaching baseline training of the highest difficulty level (stimulus duration of 1.5 s) in less than three weeks on average. Similar to all other touchscreen-based tasks, the three-choice orienting task is highly automated and easy to run and task parameters such as stimulus duration, timeout duration or the amount of reward can easily be modified if needed. Although we did not perform video analysis in our touchscreen experiments, the systems are equipped with video recorders, which can be used in combination with software like ANY-maze to track the location of the animal’s head at specific times during the task. Given the broad utilization of touchscreen-based platforms for cognitive testing, this task offers the ability to test orienting behavior across labs under identical experimental paradigms, benefiting from the numerous advantages these touchscreen systems provide (Mar et al., 2013). Touchscreen-based platforms can also be combined with other techniques such as optogenetics or electrophysiology, to manipulate or record neural activity at precise time points during a testing session (for example, just before a response is made or right after a trial is initiated), and this can be used to more accurately study the role of structures implicated in spatial attention and orienting behavior (Bussey et al., 1994, 2008; Horner et al., 2013).

### Limitations

A limitation of the present study was the small number of testing sessions performed per animal, which made it difficult to get an accurate representation of the responses to left stimuli, especially regards to the extremely low number of leftward responses after CNO injection. For example, for trials with an SOA of +1, very few animals responded toward the left at all, which made it difficult to analyze measures such as left response latency given the low number of trials and animals. Similarly, rightward responses in negative SOA trials and side responses in center-only trials were relatively infrequent. Future studies should aim to test each animal multiple times or in longer test sessions, until enough trials have been performed to accurately represent all possible response scenarios. Repeated testing in this task is quite feasible, as we show that baseline performance is stable across test sessions and pretest days. Furthermore, the utilization of transient inactivation methods such as DREADDs allows the possibility of including more testing sessions without having postlesion recovery time as a confounding factor.

In summary, the novel three-choice orienting paradigm implemented in the present study has been shown to be highly sensitive to neglect-like orienting deficits that resulted from DREADD-induced unilateral deactivation of the SC. This study provides a foundation for the application of a standardized behavioral tests in a variety of research questions centered on alteration of orienting behavior.

## Synthesis

Reviewing Editor: Laura Bradfield, University of Technology Sydney Decisions are customarily a result of the Reviewing Editor and the peer reviewers coming together and discussing their recommendations until a consensus is reached. When revisions are invited, a fact-based synthesis statement explaining their decision and outlining what is needed to prepare a revision will be listed below. The following reviewer(s) agreed to reveal their identity: Alicia Izquierdo.

The reviewers and I agree that, overall, the authors have done an excellent job of responding to all editor and reviewer queries.

However, we do ask that the authors please include a reconstruction figure of their DREADDs expression so we can observe the average spread across all sections. Although the authors suggest that there wasn’t much difference in spread among animals, we believe that it would be more rigorous and reproducible if this could be reconstructed in a figure for the reader alongside the images the authors have already included, even if it shows a great deal of overlap between animals.

I also found the manuscript revisions difficult to read with the original text that was deleted struck through. Please, when resubmitting the next version of the manuscript (to be reviewed by the editor only) could the authors delete all struck through text, and only change the font of any additional text to a red or similarly salient colour.

Specific reviewer comments are attached:

Reviewer #1:

I am satisfied that the authors have addressed all issues raised by the reviewers and editor.

Reviewer #2:

The authors were generally very responsive to my comments raised in review, but it is disappointing they did not respond to requests to provide reconstructions and better visualization of the DREADDs expression. It is not standard to provide sections of 2 brains of least and greatest spread. The sections the authors do provide do not make it easy to discern their target. If doing the reconstruction is not possible, perhaps authors can instead cite evidence that this visualization of DREADDs has been conducted this way before.
