# Supplementary material for: A Novel Three-Choice Touchscreen Task to Examine Spatial Attention and Orienting Responses in Rodents
Source: eNeuro. 2021 Jul 9;8(4):ENEURO.0032-20.2021. doi: 10.1523/ENEURO.0032-20.2021 (PMC8272401; doi:10.1523/ENEURO.0032-20.2021)
Supplement: Extended Data 1 — ABET II Touch software (Lafayette Instruments). Download Extended Data 1, ZIP file. [file enu-eN-MNT-0032-20-s01.zip › Instructions for Data Analysis.docx]

| **Identifier** | **Trial Type** |
| --- | --- |
| T1 | Left trial (training only) |
| T3 | Center Trial |
| T5 | Right trial (Training only) |
| T6 | SOA = 0 |
| T7 | SOA = -1 |
| T8 | SOA = -0.5 |
| T9 | SOA = +0.5 |
| T10 | SOA = +1 |

**Response types**

C1-C5 indicate which panel the animal responded to

**Latency measures**

The prefix L- indicates this is the average latency for that particular response.

For example, L-T3-C5 indicates the average latency for an animal that chose the rightmost panel (C5) in a center trial (T3)

Because the average is dependent on the number of trials, L-T3-C5-Count indicates the number of T3 trials where an animal pressed C5. That way, a weighted latency measure can be calculated from different days (e.g if a rat did 5 such responses on day 1 but only such responses on day 2, then the values from day 1 have a bigger effect on the overall mean across days 1 and 2)

**Calculation of values**

Response percentage is usually calculated as a fraction of total response trials

Omission percentage is calculated as a fraction of total trials

For example, if an animal had 10 T3 trials. Two of the 10 trials were omissions, therefore omission percentage is 2/10*100=20%

Out of the remaining 8 trials, the animal chose C5 four times, in which case the C5 percentage is 4/8*100=50%

**IR Beam measures**

These were not included in the data but are indicators of where the animal's body was during 1. stimulus presentation and 2. when an omission is counted

Front beam (FIR) is on the end where the touch screen is

Back beam (BIR) is on the end where the food magazine is

FIR_ON_BIR_OFF indicates that the animal was positioned near the screen (crossing the front beam but not the back beam)

FIR_OFF_BIR_ON indicates that the animal was positioned near the food magazine

FIR_OFF_BIR_OFF indicates that the animal was in the middle of the chamber, not crossing either IR beams

FIR_ON_BIR_ON indicates that the animal was large or that its head was crossing one beam and its tail was crossing the other beam

Latency tray to BIR OFF indiciates the time it took the animal to turn around and move away from the back of the chamber, after it nose poked into the food magazine (typically initiating a trial)

Latency tray to FIR ON indicates the time it took for the animal to turn around and move to the front of the chamber, where the touch screen is, after it nose poked into the food magazine (typically initiating a trial)
